# An updated insight into the effect of β-adrenergic receptor antagonists (β-blockers) on respiratory function in asthma patients: a systematic review and meta-analysis

**DOI:** 10.3389/fphys.2025.1582740

**Published:** 2025-07-25

**Authors:** Monika Marko, Rafał Pawliczak

**Affiliations:** Department of Immunopathology, Faculty of Medicine, Division of Biomedical Science, Medical University of Lodz, Lodz, Poland

**Keywords:** asthma, beta-blockers, cardio-selective, FEV1, non-selective, PEFR, safety

## Abstract

**Background:**

This study aimed to provide an updated assessment of the changes in respiratory function after β-adrenergic receptor antagonists (β-blockers) administration in asthma patients. The main assumption of the study was to use new methodological and statistical approaches not previously applied in this field in systematic reviews and meta-analyses.

**Methods:**

To select studies, PubMed/Medline, Embase, ClinicalTrials.gov, and Cochrane Library were searched. Additionally, Google Scholar was searched for gray literature. A systematic review and meta-analysis for forced expiratory volume in 1 second (FEV_1_) and peak expiratory flow rate in asthma patients after administration of cardio-selective and non-selective β-blockers compared to placebo or baseline was performed. We also assessed FEV_1_ after topical β-blocker application compared to baseline.

**Results:**

An independent subgroup analysis demonstrated significantly higher FEV_1_ in the placebo group (standardized mean difference [SMD] =−0.74, 95% confidence interval [CI]: 1.15, −0.34, P = 0.0003) than in non-selective β-blockers. The test for subgroup differences indicates that there is a statistically significant subgroup effect among cardio-selective and non-selective β-blockers (P = 0.03, *I* ^2^ = 80%). We also showed a statistically significant decrease in FEV_1_ (SMD = −0.70, 95% CI: [−1.56 to −0.03], P = 0.04) after topical β-blocker application.

**Conclusion:**

Patients with asthma may tolerate cardio-selective β-blockers better than non-selective β-blockers. The FEV1 value depends on the type of β-blocker used. Cardio-selective β-blockers may be cautiously considered in patients with asthma only when strong cardiovascular indications exist (such as heart failure with reduced ejection fraction or post-myocardial infarction) and with appropriate monitoring. At the same time, less risky therapeutic options should be chosen instead of topical β-blockers.

**Systematic Review Registration:**

https://www.crd.york.ac.uk/prospero/, identifier 42024606876.

## 1 Introduction

According to the Global Initiative for Asthma (GINA) Main Report (GINA, 2024), asthma is a heterogeneous condition commonly involving persistent inflammation of the airways. It is characterized by a pattern of symptoms, including wheezing, breathlessness, coughing, and chest tightness, which fluctuate in intensity and frequency over time. These symptoms are typically accompanied by reversible or variable limitation of airflow during exhalation, which can be confirmed through pulmonary function tests (PFTs). PFTs support the diagnosis by demonstrating reversible or variable airflow obstruction, most defined as an increase in FEV_1_ of ≥12% and ≥200 mL after administration of a bronchodilator. Larger variability, such as an increase in FEV_1_ of >12% and ≥400 mL or significant changes observed between visits or after anti-inflammatory treatment, provides stronger diagnostic support (GINA, 2024; Stanojevic et al., 2022). Despite continued advances in asthma treatment and the development of current guidelines (GINA, 2024), the condition remains a significant health and socioeconomic burden (Patel and Teach, 2019). Moreover, various comorbidities often play a role in the course, severity, and control of asthma (Rogliani et al., 2022; Tiotiu et al., 2019). In particular, attention is paid to suggested links between cardiovascular diseases (CVDs) and asthma morbidity (Tiotiu et al., 2019; Cazzola et al., 2022), the likelihood of which is up to three times higher than in the general population, impairing optimal asthma control (Tiotiu et al., 2019). In practice, this co-occurrence poses a significant challenge because drugs used successfully in CVDs, including β-adrenergic receptor antagonists (β-blockers), have been shown to be associated with increased airway hyperresponsiveness (AHR) and airflow limitation in patients with asthma, as well as in the general population (Tiotiu et al., 2019). This, in turn, can lead to an exacerbation in asthma patients or the appearance of asthma symptoms (Tiotiu et al., 2019; Morales et al., 2011; Morales et al., 2014). Because of this decade-old belief, β-blockers are not recommended for use in asthma (Salpeter et al., 2002). In addition to being commonly used in the treatment of CVDs such as congestive heart failure, ischemic heart disease, cardiac arrhythmias, and hypertension (Huang et al., 2021; Bennett M. et al., 2021), β-blockers can help reduce systemic inflammation, decrease the number of goblet cells, and limit mucus production. Therefore, even in the absence of obvious cardiac conditions, β-blockers may be advantageous for individuals with chronic obstructive pulmonary disease COPD(12).

β-blockers are a group of drugs that block β-adrenoreceptors. There are three main types of β-adrenoreceptors, divided according to their distribution: β1 (predominant in the heart), β2 (present in bronchial and vascular smooth muscle, skeletal muscle, uterus, and liver), and β3 (found in adipose tissue) (Tiotiu et al., 2019). However, the most discussed issues concern the β1 and β2 receptors. There are still no clinical studies that establish the therapeutic and clinical use of blockers that affect the β3 receptors (Samanta et al., 2024). β-blockers vary in their selectivity for these receptors. A distinction was made between β1-selective β-blockers (the cardio-selective atenolol, metoprolol, bisoprolol, nebivolol, carvedilol, and esmolol) and non-selective β-blockers, blocking β1 receptors in the heart and β2 receptors in airways, blood vessels, and liver (propranolol, nadolol, timolol, and sotalol) (Tiotiu et al., 2019; Kuipers et al., 2018).

The introduction of cardioselective β-blockers aimed to preferentially block cardiac β1-adrenergic receptors with less activity at airway β2-adrenergic receptors (Bennett M. et al., 2021). It has been demonstrated that, in therapeutic doses, cardioselective β-blockers exert β2-antagonism, but to a lesser extent than non-selective β-blockers. However, it has been shown that cardioselective β-blockers do not lead to an increase in asthma exacerbations, decrease airway function, or deteriorate the quality of life in patients with asthma and cardiovascular comorbidity (Tiotiu et al., 2019). Although cardio-selective β-blockers may be safer than non-selective β-blockers, the issue of risk assessment for the use of cardio-selective β-blockers in patients with asthma has not been fully resolved (Bennett M. et al., 2021). The reason may be that the previous theory, which suggested the need to completely exclude β-blockers in patients with an underlying disease of concern to adverse events, such as asthma, diabetes mellitus, and peripheral artery disease, is still remembered (Huang et al., 2021). It has also been shown that the administration of topical β-blockers, similar to systemic β-blockers, may cause a worsening of respiratory function (Dunn et al., 1986; Diggory et al., 1993) and exacerbate asthma attacks in glaucoma patients with asthma (Kido et al., 2022).

There are also still no large studies assessing the risks associated with the use of β-blockers in patients with asthma in real-life settings (Tiotiu et al., 2019). As a result, in everyday practice, many physicians still avoid prescribing cardio-selective β-blockers in patients with asthma. This, in turn, may be a limitation for these patients as they are deprived of the benefits of β-blocker therapy in relation to CVDs or other conditions requiring the introduction of this type of medication. Although these issues have been considered for a long time, this problem continues to pose challenges for practitioners (Bennett M. et al., 2021) who are faced with the dilemma of whether to administer a β-blocker to an asthma patient and which one will be the safest.

In this study, a systematic review and meta-analysis was conducted to assess the safety of β-blockers and evaluate the changes in respiratory function after β-blocker administration in asthma patients. An attempt was made to detect whether cardio-selective β-blockers are better tolerated than non-selective β-blockers. An additional objective was to assess which β-blocker is least associated with decreased respiratory function in patients with asthma.

Previously published systematic reviews and meta-analyses (Morales et al., 2014; Huang et al., 2021; Bennett M. et al., 2021) on the safety of β-blockers in asthma have not fully resolved this dilemma. Therefore, in this study, we decided to perform a systematic review and meta-analysis that would provide a new approach to the issue by using methods and interpretations different from those in currently available publications. Our goal was to consider new outcomes and introduce measures of effects that had not been previously applied. In addition, we employed various analytical methods, including subgroup analysis, which may provide updated conclusions and new insights into the issue.

It should be emphasized that to obtain reliable results in our analytical approach, we did not analyze cardio-selective β-blockers and non-selective β-blockers together in a single subgroup or analysis but instead separated them. In addition, we took up the challenge of selecting studies that described patients using β-mimetics (such as salbutamol) and β-blockers, which may also have a significant impact on the interpretation of the results, a consideration that has not been made before.

## 2 Methods

### 2.1 Search strategy and selection criteria

A systematic review and meta-analysis was performed according to Preferred Reporting Items for Systematic Reviews and Meta-Analyses (PRISMA) (Page et al., 2021). The study protocol was described in PROSPERO (ID: CRD42024606876). PubMed/Medline, Cochrane Central Register of Controlled Trials (CENTRAL), Embase, and ClinicalTrials.gov were searched through October 2024. Subsequently, gray literature (including conference proceedings, published reports, non-peer-reviewed publications, or datasets, white papers, and patents) was searched by using Google Scholar. The search strategy is presented in Table 1. To identify further studies for possible inclusion, searches were repeated before the final analysis.

**TABLE 1:** Search strategy.

Database	Search strategy
PubMed/Medline	((beta-adrenergic blocking agents) OR (beta-adrenergic blocking drugs) OR (beta-adrenergic receptor antagonists) OR (beta-adrenergic receptor blockers) OR (beta-adrenoceptor blockade) OR (beta-blockade) OR (adrenergic beta-antagonists) OR (beta-blockers) OR (beta-blocker) OR (beta1-blockers) OR (beta1-blocking) OR (acebutolol) OR (adaprolol) OR (adimolol) OR (alprenolol) OR (amosulalol) OR (ancarolol) OR (arnolol) OR (arotinolol) OR (atenolol) OR (befunolol) OR (betaxolol) OR (bevantolol) OR (bisoprolol) OR (bometolol) OR (bopindolol) OR (bornaprolol) OR (bromoacetylalprenololmenthane) OR (bucindolol) OR (bucumolol) OR (bufetolol) OR (bufuralol) OR (bunitrolol) OR (bunolol) OR (bupranolol) OR (butofilolol) OR (butoxamine) OR (carazolol) OR (carteolol) OR (carvedilol) OR (celiprolol) OR (cetamolol) OR (cicloprolol) OR (cloranolol) OR (dexpropranolol) OR (diacetolol) OR (epanolol) OR (esmolol) OR (exaprolol) OR (falintolol) OR (flestolol) OR (flusoxolol) OR (indenolol) OR (labetalol) OR (landiolol) OR (levobetaxolol) OR (levobunolol) OR (levomoprolol) OR (medroxalol) OR (mepindolol) OR (metipranolol) OR (metoprolol) OR (moprolol) OR (nadolol) OR (nadoxolol) OR (nebivolol) OR (nifenalol) OR (nipradilol) OR (oxprenolol) OR (pacrinolol) OR (pafenolol) OR (pamatolol) OR (pargolol) OR (penbutolol) OR (pindolol) OR (practolol) OR (primidolol) OR (prizidilol) OR (procinolol) OR (propranolol) OR (ridazolol) OR (ronactolol) OR (soquinolol) OR (sotalol) OR (spirendolol) OR (talinolol) OR (tazolol) OR (tertatolol) OR (tienoxolol) OR (tilisolol) OR (timolol) OR (tiprenolol) OR (tolamolol) OR (xibenolol)) AND ((safety) OR (adverse events) OR (adverse effects) OR (asthma exacerbation) OR (asthma) OR (asthmatic) OR (respiratory disease) OR (airway disease) OR (wheeze) OR (wheezing) OR (dyspnea) OR (bronchial hyperreactivity) OR (bronchial constriction)) Filters applied: Clinical Trial
Cochrane Central Register of Controlled Trials (CENTER)	((beta-adrenergic blocking agents) OR (beta-adrenergic receptor antagonists) OR (beta blockers)) AND ((safety) OR (adverse events) OR (adverse effects) OR (asthma exacerbation) OR (asthma)) Filters applied: title abstract keyword, in Trials
ClinicalTrials.gov	((beta-adrenergic blocking agents) OR (beta-adrenergic receptor antagonists) OR (beta blockers)) AND ((safety) OR (adverse events) OR (adverse effects) OR (asthma exacerbation) OR (asthma)) Filters applied: In “Expert search”
Embase	((beta-adrenergic blocking agents) OR (beta-adrenergic receptor antagonists) OR (beta blockers)) AND ((safety) OR (adverse events) OR (adverse effects) OR (asthma exacerbation) OR (asthma))
Google Scholar	((beta-adrenergic blocking agents) OR (beta-adrenergic receptor antagonists) OR (beta blockers)) AND ((safety) OR (adverse events) OR (adverse effects) OR (asthma exacerbation) OR (asthma))

The research question and selection criteria were formulated using the Population, Intervention, Comparison and Outcome (PICO) structure (Cumpston et al., 2020). Additionally, study type (T) has been added to the PICO framework. The inclusion criteria were as follows: (a) population: patients with asthma or with asthma and conditions subject to therapy with β-blockers; (b) intervention: β-blockers; (c) comparison: placebo or baseline or another β-blocker; (d) outcomes: forced expiratory volume in 1 second (FEV_1_), peak expiratory flow rate (PEFR), and/or number of incidences such as asthma exacerbation, wheezing, dyspnea, bronchial hyperreactivity, bronchial constriction and asthma attacks. It was determined that the literature would be searched for randomized clinical trials (RCTs), non-randomized clinical trials, real-life trials, observational trials, open-label trials, and prospective trials.

The following exclusion criteria were formulated: (a) review article; (b) systematic reviews; (c) meta-analysis; (d) case series; (e) case report; (f) articles with insufficient information and data; (g) articles published in languages other than English; (h) original articles where specific data and outcomes could not be extracted; (i) original articles that do not include outcomes of interest; (j) retracted articles.

### 2.2 Study selection and data extraction

The research selection and data extraction processes were carried out in several stages by two researchers independently at the same time. First, abstracts and titles of articles available in the databases were reviewed. Duplicates were rejected from the pool of obtained articles. In the next step, each selected article underwent a full-text review to ensure it met the inclusion criteria. To minimize the potential risk of selection bias, investigators independently assessed whether to reject or include the study.

In the next stage, the data extraction process was performed independently by each investigator. Then, the data were cooperatively reviewed. If there were divergent views regarding the classification of results, negotiations were held until a consensus was reached. Each researcher collected information about studies and data in separate sheets (titles, authors, and institutions, study design, duration of the study, publication date, study identification/registration number, intervention, the number and age of participants, disease, and outcomes). Subsequently, the content of the sheets was compared in terms of details and characteristics of selected studies. Moreover, selected studies were screened for missing or unclear data and information. According to Cochrane standards (Higgins et al., 2024), data presented as a confidence interval (CI) or standard error of the mean (SEM) were converted to mean and standard deviation (SD).

### 2.3 Assessment of the risk of bias and methodological quality

The risk of bias assessment tool in the Cochrane Review Manager 5.4 software was used to assess the methodological quality and risk of bias. The investigators judged individually included studies as “low risk of bias,” “unclear risk of bias,” and “high risk of bias” for seven items (random sequence generation, allocation concealment, blinding of participants and personnel, blinding of outcome assessment, incomplete outcome data, selective reporting, and other bias). A possible conflict of interest was checked due to the relationship between the results and the funding sources in the included studies. The researchers considered divergent opinions during the discussion. The allowable value of losses influencing the research results was set at 10%.

### 2.4 Statistical methods

The obtained data were collected in a database and analyzed quantitatively using Cochrane Review Manager 5.4 software. The results comprised continuous data displayed as standardized mean difference (SMD) with 95% CI in each group. Because heterogeneity was suspected after including the studies in the meta-analysis, the Mantel–Haenszel test with a random-effects model was used for all results. This approach was designed to minimize the risk of bias in selecting effect sizes and to optimally utilize extracted data from studies that varied in participant composition and clinical heterogeneity. Because most outcomes are presented in different units (different dosages of β-blockers), mean differences were converted into standardized mean differences (SMDs). Cochran’s Q test and I square (*I* ^2^) indices were used to evaluate heterogeneity among the outcomes of the studies included in the meta-analysis. The results were considered statistically significant at P < 0.05. The results of the meta-analysis were also presented visually using forest plots. For interpretability, SMD values were classified according to Cohen’s effect size thresholds: small (approximately 0.2), medium (approximately 0.5), and large (0.8 or greater) (Andrade, 2020). Negative SMD values indicate a lower mean in the intervention group than the control group.

### 2.5 Additional analyses

The studies included in the meta-analysis (quantitative analysis) were further evaluated for the certainty of the evidence using the Grading of Recommendations Assessment, Development and Evaluation (GRADE) method (Schünemann et al., 2023; Chu et al., 2021; Guyatt et al., 2013). The quality assessment of the studies considered factors such as risk of bias, inconsistency, indirectness, imprecision, and publication bias. Based on this evaluation, the studies were categorized as having high, moderate, low, or very low certainty, according to the standardized GRADE terminology (Cochrane Effective Practice and Organisation of Care EPOC, 2017; Schumemann et al., 2013). The results of the assessment were then summarized using a Summary of Findings (SoF) table (Cochrane Effective Practice and Organisation of Care EPOC, 2017).

A sensitivity analysis was conducted to evaluate the robustness of the meta-analysis. We compared the results of the meta-analysis that included studies with a high risk of bias (identified during the risk of bias assessment) with those obtained after excluding these studies from the analysis.

In our study, fewer than 10 different studies were included in a single forest plot. Therefore, we were unable to perform a funnel plot analysis to assess publication bias because, as per Cochrane guidelines (Higgins et al., 2024), the power of this test is too low to detect fundamental asymmetry in meta-analyses with fewer than 10 studies.

## 3 Results

### 3.1 Included studies

**FIGURE 1:**
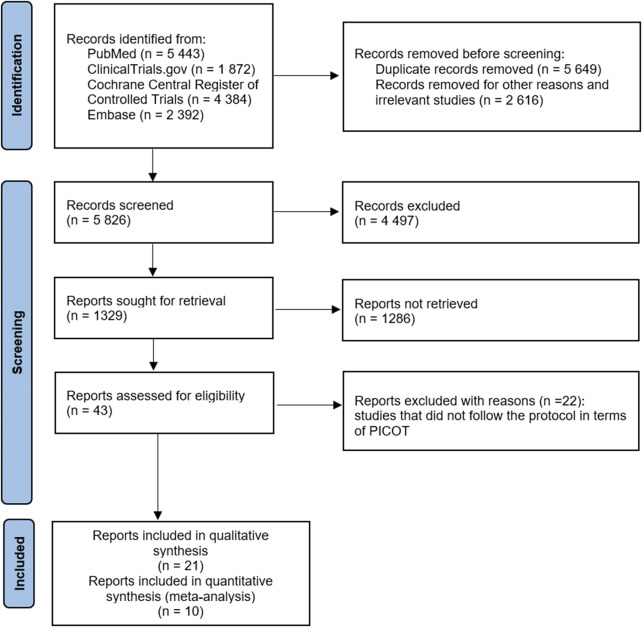
Flowchart of the screening procedure. PICOT: Population, Intervention, Comparison, Outcome and Study type.

Figure 1 (Page et al., 2021) shows that the records screened included 5,826 related articles. After full-text screening, assessment for eligibility, and quality evaluation, 21 articles meeting the inclusion criteria were included for qualitative analysis. At the same time, 22 reports were excluded for reason. The included studies were RCTs, non-randomized clinical trials, open-label trials, and retrospective and prospective trials. After quality assessment, ten studies were included for quantitative analysis (meta-analysis). Study details are shown in Table 2. Due to the lack of or too few data on the safety of β-blockers, such as the number of asthma attacks or asthma exacerbations, we could not use them in the meta-analysis as planned at the beginning of the study and described in PICOT (Population, Intervention, Comparison, Outcome and Study type). Therefore, our systematic review and meta-analysis primarily focused on outcomes related to respiratory function, such as FEV_1_ and PEFR. No literature item was included in the study at the gray literature search stage (Google Scholar), and no duplicates were found. After re-running the search, no additional studies were included in the systematic review and meta-analysis.

**TABLE 2:** Characteristics of studies included in the systematic review and meta-analysis.

Study ID	Study design	Intervention	Sample size	Age	Disease	Outcome included in systematic review and/or meta-analysis
Bennett M. R. et al. (2021)	A randomized, double-blind, placebo-controlled crossover study	Bisoprolol 5 mg, Placebo	19	18–71	Mild or moderate asthma	FEV_1_
Benson et al. (1978)	A single-blind, randomized crossover study	Propranolol 100 mg Pindolol 5 mg Atenolol 100 mg Acebutolol 300 mg Placebo	14	17–67	Asthma	FEV_1_
Decalmer et al. (1978)	A single-blind, randomized crossover study	Cardio-selective β-blockers: atenolol (100 mg), metoprolol (100 mg), and acebutolol (300 mg) non-selective β-blockers: propranolol (100 mg), oxprenolol (100 mg), pindolol (5 mg), timolol (10 mg) (2 h) Placebo	10	17–65	Asthma	FEV_1_
Ellis et al. (1981)	Double-blind, randomized, placebo-controlled study	Atenolol 50 mg, 100 mg, and 200 mg Propranolol 40 mg (2 h)	10	20–72	Asthma	FEV_1_
Greefhorst and van Herwaarden (1981)	Double-blind, placebo-controlled study	Atenolol 100 mg Metoprolol 100 mg Acebutolol 400 mg (2 h) Placebo	8	20–44	Asthma	FEV_1_ PEFR
Hanania et al. (2008)	A prospective, open-label study	Nadolol 10 mg, 20 mg, and 40 mg	10	18–50	Mild asthma	FEV_1_
Hepsen et al. (2004)	A prospective and single-masked study	Timolol eye drops	14	23–70	Mild-to-moderate asthma and normal ocular examination	Timolol-induced bronchoconstriction in asthmatics
Lammers et al. (1984)	Double-blind, placebo-controlled study	Bisoprolol 10 mg Bisoprolol 20 mg Metoprolol 100 mg (2 h) Placebo	8	24–52	Asthma	FEV_1_ PEFR
Lawrence et al. (1982)	A single-blind, randomized crossover study	Atenolol 100 mg Metoprolol 100 mg Placebo (1.5–2 h)	14	39–69	Asthma	FEV_1_ PEFR
Kido et al. (2022)	A retrospective longitudinal cohort study	Topical β-blocker users and non-β-blocker users	30,275	20–70	Glaucoma patients with asthma	The incidence of asthma attacks
Morales et al. (2016)	A population-based study	Ocular β-blocker	4865	Mean age 67.8 years	Patients with asthma and ocular hypertension	Asthma exacerbations Respiratory effect
Morales et al. (2017)	A population-based nested case–control study	Cardio-selective and non-selective β-blockers prescribed in asthma and CVD	35,502	Mean age 60.1 years	Asthma and CVDs	Moderate and severe asthma exacerbations
Myers et al. (1996)	A randomized, crossover, double-blind, placebo-controlled design	Propranolol from 0.25 mg/mL to 32 mg/mL	12	22–45	Stable mild asthma	Propranolol-induced bronchoconstriction
P Philip-Joet et al. (1986)	A double-blind study	Bevantolol 400 mg Atenolol 100 mg	7	Mean age 40.8 ± 4.8	Asthma	FVC_1_
Ruffin et al. (1982)	A double-blind, crossover study	Propranolol 40 mg and 160 mg Pindolol 5 mg and 20 mg Atenolol 50 mg and 200 mg	12	21–37	Mild-to-moderate asthma, with some patients requiring regular inhaled bronchodilator or beclomethasone	FEV_1_
Schindl et al. (1986)	A double-blind, randomized, crossover study	Atenolol (100 mg) Celiprolol (200 mg) Metoprolol (200 mg)	16	43–75	Asthma and hypertension	FEV_1_
Schoene et al. (1984)	A randomized, double-masked, crossover study	Topical betaxolol 1% and timolol 0.5%	9	25–69	Patients with asthma or asthmatic bronchitis	Pulmonary function
S Short et al. (2012)	A *post hoc* analysis of a randomized placebo-controlled crossover study	Propranolol 10 mg and 20 mg	13	19–63	Mild-to-moderate persistent stable asthma	Spirometry in assessing bronchoconstriction to propranolol and bronchodilation with salbutamol
Short et al. (2013)	A double-blind randomized placebo-controlled crossover trial	Propranolol	18	19–65	Mild-to-moderate asthma	FEV_1_
Short et al. (2014)	A *post hoc* analysis of a double-blind, randomized, placebo-controlled trial	Esmosol 0.5 mg/kg Propranolol 10 mg, 20 mg, and 80 mg	12	19–65	Mild-to-moderate asthma	FEV_1_
Dunn et al. (1986)	A clinical trial	Topical timolol Topical betaxolol	24	20–66	Asthma or a history compatible with reactive airway disease	FEV_1_

Abbreviations: FEV_1_, forced expiratory volume in 1 s; FVC, forced vital capacity; CVD, cardiovascular diseases; PEFR, peak expiratory flow rate.

### 3.2 Assessment of methodological quality

**FIGURE 2:**
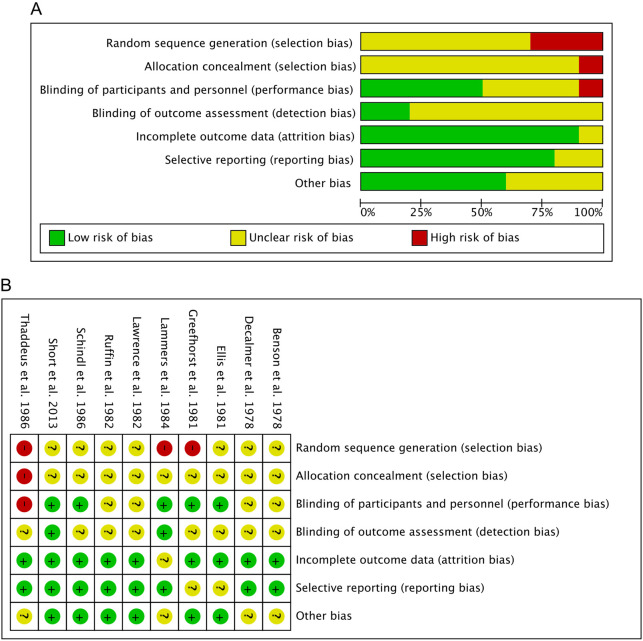
Risk of bias assessment. **(A)** Risk of bias summary: Review authors’ judgements about each risk of bias item for each included study. **(B)** Risk of bias graph: Review authors’ judgments about each risk of bias item are presented as percentages across all included studies.

Our comprehensive evaluation process of studies qualified for meta-analysis included ten studies for assessing the risk of bias. The assessment of methodological quality revealed that for random sequence generation, three (30.00%) of the included studies had a high risk of bias, and seven (70.00%) studies showed an unclear risk. For allocation concealment, one (10.00%) of the included studies had a high risk of bias. The remaining nine (90.00%) studies showed an unclear risk of these biases. For performance bias, one (10.00%) of the included studies had a high risk of bias, four (40.00%) studies had an unclear risk of bias, and five (50.00%) studies had a low risk of bias. For detection bias, two (20.00%) studies had a low risk of bias. The remaining eight (80.00%) studies showed an unclear risk of these biases. For attrition bias, nine studies (90.00%) showed a low risk of bias, and one (10.00%) study had an unclear risk of bias. For reporting bias, eight (80.00%) studies had a low risk of bias. In contrast, the remaining two (20.00%) studies showed an unclear risk of bias. In case of other biases, six (60.00%) studies showed a low risk of bias, and four (40.00%) studies showed an unclear risk of bias. The results obtained from the methodological evaluation are presented in Figures 2A,B. We did not detect strong evidence of publication bias following a qualitative assessment of the included studies. Because independent analyses in our study included fewer than 10 studies, according to Cochrane recommendations (Higgins et al., 2024), we did not provide a funnel plot to assess publication bias, as the power of this test is too low to distinguish true asymmetry in these cases.

### 3.3 Cardio-selective and non-selective β-blockers: FEV_1_

Six original articles (Benson et al., 1978; Decalmer et al., 1978; Ellis et al., 1981; Greefhorst and van Herwaarden, 1981; Lammers et al., 1984; Lawrence et al., 1982) were eligible for subgroup meta-analysis for FEV_1_ results after β-blocker administration compared to placebo. Studies were divided into two or three parts because the authors presented the results for different treatment dosages. The studies were divided into two subgroups, including cardio-selective β-blockers and non-selective β-blockers.

The polled data included 186 patients in the experimental group and 206 patients in the placebo group. Independent subgroup analysis demonstrated significantly higher FEV_1_ in the placebo group (standardized mean difference [SMD] = −0.74, 95% confidence interval [CI]: [ 1.15, −0.34], P = 0.0003, *I* ^2^ = 0%, P = 0.96) than in the non-selective β-blocker group. According to Cohen’s thresholds (Andrade, 2020), the observed SMD of −0.74 represents a medium to large effect size, indicating a clinically meaningful reduction in FEV_1_ in the group receiving non-selective β-blockers compared to placebo. This finding supports the conclusion that non-selective β-blockers are associated with a substantial decline in pulmonary function in patients with asthma.

In the subgroup of cardio-selective β-blockers, we also observed better FEV_1_ outcomes in the placebo group, although without statistical significance (SMD = −0.21, 95% CI: [−0.44 to 0.03], P = 0.08, *I* ^2^ = 0%, P = 1.00). Although the effect did not reach statistical significance (P > 0.05), the observed SMD of −0.21 corresponds to a small effect size according to Cohen’s criteria (Andrade, 2020). The difference may still be clinically relevant, although the result should be interpreted with caution due to limited statistical power.

**FIGURE 3:**
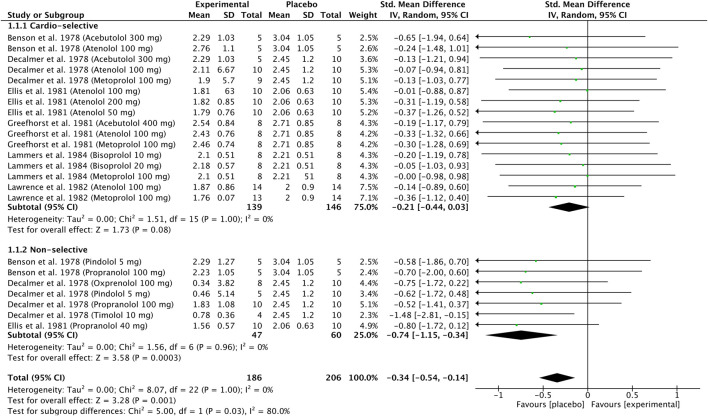
Standardized mean difference (SMD) for FEV_1_. Cardio-selective and non-selective β-blockers. FEV_1_: forced expiratory volume in 1 second.

However, the test for the subgroup overall effect showed a significantly higher FEV_1_ (SMD = −0.34, 95% CI: [−0.54 to −0.14], P = 0.001, *I* ^2^ = 0%, P = 1.00) in the placebo group. Consistent with Cohen’s thresholds (Andrade, 2020), the observed SMD of −0.34 represents a small to medium effect size. Furthermore, the test for subgroup differences indicates a statistically significant subgroup effect (P = 0.03, *I* ^2^ = 80%). Consequently, we can state that the type of β-blocker (cardio-selective or non-selective) has a clinically relevant impact on FEV1 results in patients with asthma. The results are shown in Figure 3.

### 3.4 Non-selective β-blockers in patients using salbutamol: FEV_1_

**FIGURE 4:**
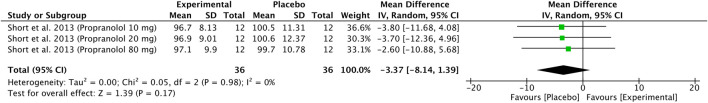
Standardized mean difference (SMD) for FEV_1_. Non-selective β-blockers in patients using salbutamol. FEV_1_: forced expiratory volume in 1 second.

One original article (Short et al., 2014) was eligible for meta-analysis for FEV_1_ results after β-blocker administration compared to placebo in asthma patients using salbutamol. This study was divided into three parts because the authors presented the results for different treatment dosages. Polled data provided 36 patients in the experimental group and 36 patients in the placebo group. The meta-analysis showed that despite inhaled therapy, patients in the β-blocker group had lower FEV_1_ than placebo but with no statistical significance (SMD = −3.37, 95% CI: (−8.14 to 1.39), P = 0.17, *I* ^2^ = 0%, P = 0.98). Despite the lack of statistical significance (P > 0.05), the SMD of −3.37 reflects a large effect size based on Cohen’s criteria (Andrade, 2020), indicating potential clinical relevance that warrants cautious interpretation given the limited power. The results are shown in Figure 4.

### 3.5 Cardio-selective β-blocker baseline and after treatment: FEV_1_

**FIGURE 5:**
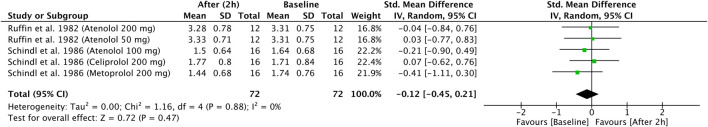
Standardized mean difference (SMD) for FEV_1_. Cardio-selective β-blockers baseline and after treatment. FEV_1_: forced expiratory volume in 1 second.

Two original articles (Ruffin et al., 1982; Schindl et al., 1986) were eligible for meta-analysis for FEV_1_ results after cardio-selective β-blocker administration compared to baseline. Studies were divided into two or three parts because the authors presented the results for different treatment dosages. Polled data provided 72 patients in the experimental group and 72 patients in the baseline. The meta-analysis showed that patients 2 hours after β-blocker administration had slightly lower FEV_1_ than baseline but with no statistical significance (SMD = −0.12, 95% CI: [−0.45 to 0.21], P = 0.47, *I* ^2^ = 0%, P = 0.88). The observed SMD of −0.12 represents a very small effect size, according to Cohen’s classification (Andrade, 2020), where values below 0.2 are generally considered negligible in terms of clinical impact. The confidence interval (−0.45 to 0.21) crosses zero, and the result is not statistically significant (P = 0.47), suggesting a high degree of uncertainty regarding the presence of any true effect. Although the analysis showed no statistical heterogeneity (*I* ^2^ = 0%), the overall findings indicate that any potential difference between groups is likely minimal and not clinically meaningful. This result should be interpreted as inconclusive and likely reflects either a true lack of effect or insufficient sensitivity of the available data to detect a small but real difference. The results are shown in Figure 5.

### 3.6 Cardio-selective β-blockers compared to placebo: PEFR

**FIGURE 6:**
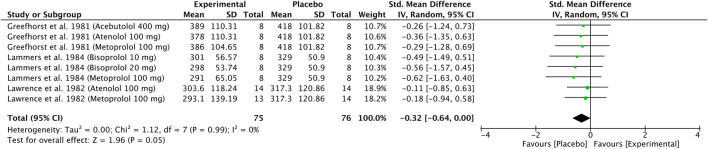
Standardized mean difference (SMD) for PEFR. Cardio-selective β-blockers compared to placebo. PEFR: peak expiratory flow rate.

Three original articles (Greefhorst and van Herwaarden, 1981; Lammers et al., 1984; Lawrence et al., 1982) were eligible for meta-analysis for PEFR results after administration of cardio-selective β-blockers compared to placebo. Studies were divided into two or three parts because the authors presented the results for different treatment dosages. The polled data provided 75 patients in the experimental group and 76 patients in the placebo group. The meta-analysis showed that patients after β-blocker administration had lower PEFR than placebo, with a result close to statistical significance (SMD = −0.32, 95% CI: [−0.64 to 0.00], P = 0.05, *I* ^2^ = 0%, P = 0.99). The observed SMD of −0.32 represents a small to moderate effect size according to Cohen’s criteria (Andrade, 2020), where values around 0.2 are considered small and 0.5 moderate. The confidence interval (−0.64 to 0.00) includes the null value at its upper bound, and the result reaches the threshold of statistical significance (P = 0.05). Although the lack of heterogeneity (*I* ^2^ = 0%) suggests consistent findings across studies, the result should be interpreted with caution due to the borderline significance and the fact that the confidence interval just touches zero. Nevertheless, the magnitude of the effect suggests a potentially meaningful reduction in the outcome variable in the intervention group, which may have clinical relevance depending on the context and population. The results are shown in Figure 6.

### 3.7 β-blocker comparisons: Baseline and after treatment

#### 3.7.1 Atenolol and acebutolol: FEV_1_

**FIGURE 7:**
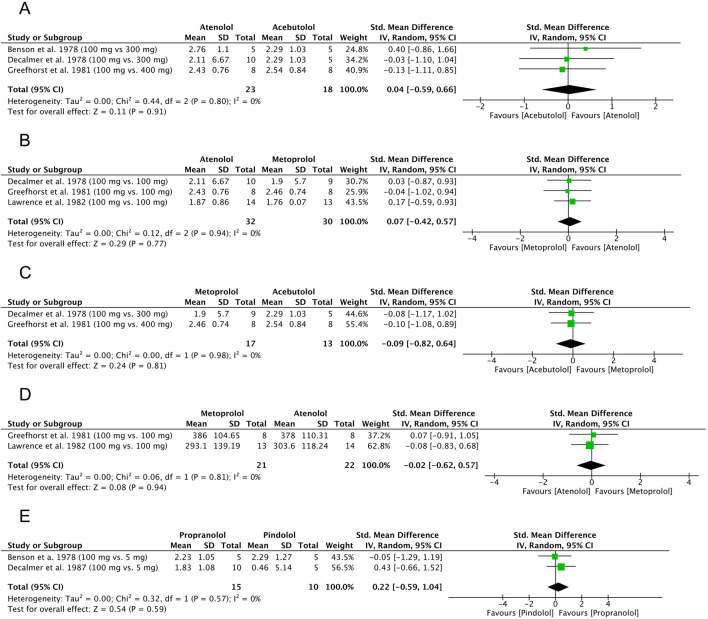
Standardized mean difference (SMD) for FEV_1_ and PEFR. **(A)** β-blocker comparison: atenolol and acebutolol, FEV_1_. **(B)** β-blocker comparison: atenolol and metoprolol, FEV_1_. **(C)** β-blocker comparison: metoprolol and acebutolol, FEV_1_. **(D)** β-blocker comparison: atenolol and metoprolol, PEFR. **(E)** β-blocker comparison: propranolol and pindolol, FEV_1_. FEV_1_: forced expiratory volume in 1 second, PEFR: peak expiratory flow rate.

Three original articles (Guyatt et al., 2013; Cochrane Effective Practice and Organisation of Care EPOC, 2017; Benson et al., 1978) were eligible for meta-analysis for FEV_1_ results after administration of atenolol compared to acebutolol. The polled data provided 23 patients in the atenolol group and 18 patients in the acebutolol group. The meta-analysis revealed that patients in both groups had similar FEV_1_ levels after β-blocker administration, although this difference was not statistically significant (SMD = 0.04, 95% CI: [−0.59 to 0.66], P = 0.91, *I* ^2^ = 0%, P = 0.80). The observed SMD of 0.04 corresponds to a negligible effect size according to Cohen’s criteria (Andrade, 2020), where values below 0.2 are generally considered clinically unimportant. The 95% confidence interval (−0.59 to 0.66) is wide and includes zero, indicating a high degree of uncertainty and no statistically significant difference between groups (P = 0.91). Additionally, the absence of heterogeneity (*I* ^2^ = 0%) suggests consistency across studies, but the data overall point to no meaningful clinical effect. This result is best interpreted as a true null or near-null finding, with no indication of benefit or harm attributable to the intervention. The results are shown in Figure 7A.

#### 3.7.2 Atenolol and metoprolol: FEV_1_

Three original articles (Decalmer et al., 1978; Greefhorst and van Herwaarden, 1981; Lawrence et al., 1982) were eligible for meta-analysis for FEV_1_ results after administration of atenolol compared to metoprolol. The polled data provided 32 patients in the atenolol group and 30 patients in the acebutolol group. The meta-analysis revealed that patients in the metoprolol group had a slightly lower FEV1 after β-blocker administration, but the difference was not statistically significant (SMD = 0.07, 95% CI: [−0.42 to 0.57], P = 0.77, *I* ^2^ = 0%, P = 0.94). The observed SMD of 0.07 represents a very small and clinically negligible effect size according to Cohen’s guidelines (Andrade, 2020), where values below 0.2 are generally interpreted as minimal or no effect. The wide confidence interval (−0.42 to 0.57) includes zero, and the result is not statistically significant (P = 0.77), indicating a high degree of imprecision and a lack of evidence for a meaningful difference between groups. The lack of heterogeneity (*I* ^2^ = 0%) suggests consistent findings across studies. Overall, this result points to a likely absence of clinically relevant effect. The results are shown in Figure 7B.

#### 3.7.3 Metoprolol and acebutolol: FEV_1_

Two original articles (Decalmer et al., 1978; Greefhorst and van Herwaarden, 1981) were eligible for meta-analysis for FEV_1_ results after administration of metoprolol compared to acebutolol. The polled data provided 17 patients in the metoprolol group and 13 patients in the acebutolol group. The meta-analysis showed that patients in the metoprolol group had a slightly lower FEV1 after β-blocker administration, but the difference was not statistically significant (SMD = −0.09, 95% CI: [−0.82 to 0.64], P = 0.81, *I* ^2^ = 0%, P = 0.98). The observed SMD of −0.09 corresponds to a negligible effect size, as values below 0.2 are generally considered clinically unimportant according to Cohen’s criteria (Andrade, 2020). The wide confidence interval (−0.82 to 0.64) crosses zero and includes both small-to-large negative and positive values, reflecting a high degree of imprecision. The result is not statistically significant (P = 0.81), and the absence of heterogeneity (*I* ^2^ = 0%) indicates consistency across studies. Overall, these findings suggest no reliable or clinically meaningful difference between groups, and the result is best interpreted as inconclusive due to both statistical non-significance and a minimal estimated effect size. The results are shown in Figure 7C.

#### 3.7.4 Metoprolol and atenolol: PEFR

Two original articles (Benson et al., 1978; Ellis et al., 1981) were eligible for meta-analysis for PEFR results after the administration of metoprolol compared to atenolol. The polled data included 21 patients in the metoprolol group and 22 patients in the atenolol group. The meta-analysis revealed that patients in the metoprolol group had a slightly lower PEFR after β-blocker administration, but the difference was not statistically significant (SMD = −0.02, 95% CI: [−0.62 to 0.57], P = 0.94, *I* ^2^ = 0%, P = 0.81). The observed SMD of −0.02 reflects a negligible effect size, as values close to zero and below 0.2 are typically considered clinically insignificant according to Cohen’s criteria (Andrade, 2020). The wide confidence interval (−0.62 to 0.57), which spans both negative and positive values and includes zero, along with the non-significant P-value (P = 0.94), indicates a very high level of uncertainty and no evidence of a difference between groups. The absence of heterogeneity (*I* ^2^ = 0%) suggests consistency across studies, but the data support the interpretation of a true null effect, with no clinically relevant benefit or harm attributable to the intervention. The results are shown in Figure 7D.

#### 3.7.5 Propranolol and pindolol: FEV_1_

Two original articles (Benson et al., 1978; Decalmer et al., 1978) were eligible for meta-analysis for FEV_1_ results after the administration of propranolol compared to pindolol. The polled data provided 15 patients in the propranolol group and 10 patients in the pindolol group. The meta-analysis showed that patients in the pindolol group had a slightly lower FEV1 after β-blocker administration, but the difference was not statistically significant (SMD = 0.22, 95% CI: [−0.59 to 1.04], P = 0.59, *I* ^2^ = 0%, P = 0.57). The observed SMD of 0.22 corresponds to a small effect size according to Cohen’s criteria (Andrade, 2020), where values between 0.2 and 0.5 are considered small. However, the wide confidence interval −0.59 to 1.04) crosses zero and spans from a moderate negative to a large positive effect, indicating substantial uncertainty regarding both the presence and direction of the effect. The result is not statistically significant (P = 0.59), and there was no heterogeneity (*I* ^2^ = 0%), suggesting consistency across included studies. Overall, this finding indicates a low likelihood of a clinically meaningful effect, although the imprecision prevents firm conclusions. The results are shown in Figure 7E.

### 3.8 Topical β-blocker comparison between baseline and after treatment: FEV_1_

**FIGURE 8:**
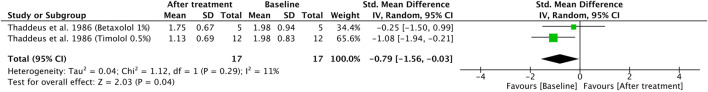
Standardized mean difference (SMD) for FEV_1_. Topical β-blocker comparison, at baseline and after FEV_1_: forced expiratory volume in 1 second.

One original article (Dunn et al., 1986) was eligible for meta-analysis for FEV_1_ results after topical β-blocker application compared to baseline. The study was divided into two parts because the author presented the results for two β-blockers (betaxolol and timolol). Polled data provided 17 patients in the experimental group and 17 patients in the baseline. The meta-analysis showed that patients after topical β-blocker application had lower FEV_1_ than baseline, with statistical significance (SMD = −0.70, 95% CI: [−1.56 to −0.03], P = 0.04, *I* ^2^ = 11%, P = 0.29). The observed SMD of −0.70 corresponds to a moderate to large effect size based on Cohen’s criteria, where values around 0.5 are considered moderate and values approaching 0.8 are considered large. The confidence interval (−1.56 to −0.03) does not cross zero, and the result is statistically significant (P = 0.04), although the wide interval suggests some degree of imprecision. The low heterogeneity (*I* ^2^ = 11%) indicates a relatively consistent effect across studies. These findings suggest a clinically meaningful reduction in the measured outcome in the intervention group compared to the control, though caution is warranted due to the borderline significance and broad confidence range. The results are shown in Figure 8.

### 3.9 Results of additional analyses

We evaluated 11 outcomes included in the meta-analysis using the certainty of the evidence (GRADE) assessment (Table 3). In the next stage, interpretations were performed based on the GRADE guidelines (Cochrane Effective Practice and Organisation of Care EPOC, 2017). This analysis showed that one outcome has high certainty of the evidence, which means that selected studies in this outcome provide a very good indication of the likely effect (the likelihood that the difference will be large enough to influence the decision is low).

**TABLE 3:** Summary of findings (SoF) of meta-analysis using Working Group Grades of Evidence (GRADE).

Patients, interventions, comparators	Participants (studies)	Quality of the evidence (GRADE)	Comparator (outcome)	Intervention vs. comparator SMD (95% CI)
Asthma patients, cardio-selective β-blockers vs. placebo	285 participants (6 studies)	⊕⊕⊖⊖ Low^a^	Mean FEV_1_	−0.21 (95% CI: 0.44 to 0.03), higher FEV_1_ in the placebo group^b^
Asthma patients, non-selective β-blockers vs. placebo	107 participants (3 studies)	⊕⊕⊕⊖ Moderate^c^	Mean FEV_1_	−0.74 (95% CI: −1.15 to −0.34), higher FEV_1_ in the placebo group^b^
Asthma patients using salbutamol, non-selective β-blockers vs. placebo	72 participants (1 study divided into 3 parts)	⊕⊕⊕⊖ Moderate^d^	Mean FEV_1_	−3.37 (95% CI: −8.14 to 1.39), higher FEV_1_ in the placebo group^b^
Asthma patients, cardio-selective β-blockers at baseline and after treatment	144 participants (2 studies)	⊕⊕⊕⊕ High^e^	Mean FEV_1_	−0.12 (95% CI: −0.45 to 0.2), higher FEV_1_ in the baseline^b^
Asthma patients, cardio-selective β-blockers vs. placebo	151 participants (3 studies)	⊕⊕⊖⊖ Low^f^	Mean PEFR	−0.32, (95% CI: −0.64 to 0.00), higher PEFR in the placebo group^b^
Asthma patients, atenolol vs. acebutolol	41 participants (3 studies)	⊕⊕⊖⊖ Low^g^	Mean FEV_1_	0.04, (95% CI: −0.59 to 0.66), The FEV_1_ values for atenolol and acebutolol were similar^b^
Asthma patients, atenolol vs. metoprolol	62 participants (3 studies)	⊕⊕⊖⊖ Low^h^	Mean FEV_1_	0.07, (95% CI: −0.42 to 0.57), lower FEV_1_ in the metoprolol group group^b^
Asthma patients, metoprolol vs. acebutolol	30 participants (2 studies)	⊕⊕⊖⊖ Low^i^	Mean FEV_1_	−0.09, (95% CI: −0.82 to 0.64), lower FEV_1_ in the metoprolol group^b^
Asthma patients, atenolol vs. metoprolol	43 participants (2 studies)	⊕⊕⊖⊖ Low^j^	Mean PEFR	−0.02, (95% CI: −0.62 to 0.57), lower PEFR in the metoprolol group^b^
Asthma patients, propranolol vs. pindolol	35 participants (2 studies)	⊕⊕⊕⊖ Moderate^k^	Mean FEV_1_	0.22, (95% CI: −0.59 to 1.04) to lower FEV_1_ in the pindolol group^b^
Asthma patients, topical β-blockers baseline vs. after	34 participants (1 study divided into 2 parts)	⊕⊖⊖⊖ Very low^l^	Mean FEV_1_	−0.70, (95% CI: −1.56 to −0.03), lower FEV_1_ after topical β-blocker application^m^

Abbreviations: 95% CI, 95% confidence interval; FEV_1_, forced expiratory volume in 1 s; GRADE, working group grades of evidence; PEFRE, peak expiratory flow rate; SMDE, standardized mean difference.

Explanations.

^a^ The evidence was downgraded from a high to low rating because of non-randomized evidence in two studies (Greefhorst and van Herwaarden, 1981; Lammers et al., 1984). The evidence was downgraded from a low to very low rating because of a risk of bias due unclear random sequence generation (Benson et al., 1978; Decalmer et al., 1978; Ellis et al., 1981; Lawrence et al., 1982), allocation concealment (Benson et al., 1978; Decalmer et al., 1978; Ellis et al., 1981; Greefhorst and van Herwaarden, 1981; Lammers et al., 1984; Lawrence et al., 1982), blinding of participants and personnel (Benson et al., 1978; Decalmer et al., 1978; Lawrence et al., 1982), blinding of outcome assessment (Benson et al., 1978; Decalmer et al., 1978; Ellis et al., 1981; Greefhorst and van Herwaarden, 1981; Lawrence et al., 1982), incomplete outcome data (Lammers et al., 1984), selective reporting (Ellis et al., 1981; Greefhorst and van Herwaarden, 1981) and other bias (Benson et al., 1978; Decalmer et al., 1978; Lammers et al., 1984). The score was then upgraded by one due to the strong association between the included outcomes and the absence of likely plausible factors.

^b^ Statistically insignificant outcome.

^c^ The evidence was downgraded from a high to a moderate rating because of a risk of bias due unclear random sequence generation (Benson et al., 1978; Decalmer et al., 1978; Ellis et al., 1981), allocation concealment (Benson et al., 1978; Decalmer et al., 1978; Ellis et al., 1981), blinding of participants and personnel (Benson et al., 1978; Decalmer et al., 1978), blinding of outcome assessment (Benson et al., 1978; Decalmer et al., 1978; Ellis et al., 1981), selective reporting (Ellis et al., 1981), and other bias (Benson et al., 1978; Decalmer et al., 1978).

^d^ The evidence was downgraded from a high to a moderate rating because of a risk of bias due to unclear random sequence generation and allocation concealment (Short et al., 2013).

^e^ The evidence was downgraded from a high to a moderate rating because of a risk of bias due to unclear random sequence generation (Ruffin et al., 1982; Schindl et al., 1986), allocation concealment (Ruffin et al., 1982; Schindl et al., 1986), blinding of participants and personnel (Ruffin et al., 1982), and blinding of outcome assessment (Ruffin et al., 1982; Schindl et al., 1986). The score was then upgraded by one due to the strong association between the included outcomes and the absence of likely plausible factors.

^f^ The evidence was downgraded from a high to a low rating because of non-randomized evidence in two studies (Greefhorst and van Herwaarden, 1981; Lammers et al., 1984). The evidence was downgraded from a low to a very low rating because of a risk of bias due unclear random sequence generation (Lawrence et al., 1982), allocation concealment (Greefhorst and van Herwaarden, 1981; Lammers et al., 1984; Lawrence et al., 1982), blinding of participants and personnel (Lawrence et al., 1982), and blinding of outcome assessment (Greefhorst and van Herwaarden, 1981; Lawrence et al., 1982). The score was then upgraded by one due to the strong association between the included outcomes and the absence of likely plausible factors.

^g^ The evidence was downgraded from a high to a low rating because of non-randomized evidence in one study (Greefhorst and van Herwaarden, 1981). The evidence was downgraded from a low to a very low rating because of a risk of bias due unclear random sequence generation (Benson et al., 1978; Decalmer et al., 1978), allocation concealment (Benson et al., 1978; Decalmer et al., 1978; Greefhorst and van Herwaarden, 1981), blinding of participants and personnel (Benson et al., 1978; Decalmer et al., 1978), and blinding of outcome assessment (Benson et al., 1978; Decalmer et al., 1978; Greefhorst and van Herwaarden, 1981). The score was then upgraded by one due to the strong association between the included outcomes and the absence of likely plausible factors.

^h^ The evidence was downgraded from a high to a low rating because of non-randomized evidence in one study (Greefhorst and van Herwaarden, 1981). The evidence was downgraded from a low to a very low rating because of a risk of bias due unclear random sequence generation (Decalmer et al., 1978; Lawrence et al., 1982), allocation concealment (Decalmer et al., 1978; Greefhorst and van Herwaarden, 1981; Lawrence et al., 1982), blinding of participants (Decalmer et al., 1978; Lawrence et al., 1982), and blinding of outcome assessment (Decalmer et al., 1978; Greefhorst and van Herwaarden, 1981; Lawrence et al., 1982). The score was then upgraded by one due to the strong association between the included outcomes and the absence of likely plausible factors.

^i^ The evidence was downgraded from a high to a low rating because of non-randomized evidence in one study (Greefhorst and van Herwaarden, 1981). The evidence was downgraded from a low to a very low rating because of a risk of bias due to unclear random sequence generation (Decalmer et al., 1978), allocation concealment, blinding of participants, and blinding of outcome assessment (Decalmer et al., 1978; Greefhorst and van Herwaarden, 1981). The score was then upgraded by one due to the strong association between the included outcomes and the absence of likely plausible factors.

^j^ The evidence was downgraded from a high to a low rating because of non-randomized evidence in one study (Greefhorst and van Herwaarden, 1981). The evidence was downgraded from a low to a very low rating because of a risk of bias due to unclear random sequence generation (Lawrence et al., 1982), allocation concealment (Greefhorst and van Herwaarden, 1981; Lawrence et al., 1982), blinding of participants and personnel (Lawrence et al., 1982), and blinding of outcome assessment (Greefhorst and van Herwaarden, 1981; Lawrence et al., 1982). The score was then upgraded by one due to the strong association between the included outcomes and the absence of likely plausible factors.

^k^ The evidence was downgraded from a high to a moderate rating because of a risk of bias due to unclear random sequence generation, allocation concealment, blinding of participants and personnel, and blinding of outcome assessment (Benson et al., 1978; Decalmer et al., 1978).

^l^ The evidence was downgraded from a high to a low rating because of non-randomized evidence. The evidence was downgraded from a low to a very low rating because of allocation concealment, blinding of participants and personnel, and unclear blinding of outcome assessment (Dunn et al., 1986).

^m^ Statistically significant outcome.

Three outcomes have moderate certainty of the evidence. These results can be interpreted as indicating that the included studies for these outcomes provide a good indication of the likely effect assessed in meta-analysis (there is a moderately probable chance that the effect will be significantly different). At the same time, six outcomes showed low certainty of the evidence. This result provides some indication of the likely effect. It should be emphasized that the probability of it being significantly different is high. One remaining outcome showed very low certainty of the evidence. In this case, one can interpret these results as not providing reliable indications of the probable effect. Additionally, there is a very high probability that the estimated effect will be significantly different.

Three studies included in the quantitative synthesis have a high risk of bias due to a lack of randomization (selection bias). Therefore, these studies were eligible for a sensitivity analysis of seven evaluated cases (Table 4). In four cases, the assessed findings were robust to sensitivity analysis, which showed slight differences in the overall effect and no change in heterogeneity values after excluding studies with a high risk of bias from the meta-analysis. In three cases, excluding studies from the meta-analysis resulted in the inability to perform a new comparison.

**TABLE 4:** Sensitivity analysis of studies included in a meta-analysis.

Outcome	Study ID	SMD [95% CI] before sensitivity analysis	SMD [95% CI] after sensitivity analysis
Asthma patients, cardio-selective β-blockers vs. placebo. Mean FEV_1_	Greefhorst and van Herwaarden (1981) Lammers et al. (1984)	−0.21 [0.44 to 0.03] P = 0.008 *I* ^2^ = 0% (P = 1.00) Test for subgroup differences: Ch*I* ^2^ = 5.00 to df = 1 (P = 0.03) *I* ^2^ = 80.0%	−0.22 [−0.51 to 0.07] P = 0.13 *I* ^2^ = 0% (P = 1.00) Test for subgroup differences: Ch*I* ^2^ = 4.20 to df = 1 (P = 0.04), *I* ^2^ = 76.2%
Asthma patients, cardio-selective β-blockers vs. placebo. Mean PEFR	Greefhorst and van Herwaarden (1981) Lammers et al. (1984)	−0.32 [−0.64 to 0.00] P = 0.05 *I* ^2^ = 0% (P = 0.99)	−0.15 [−0.67 to 0.38] P = 0.59 *I* ^2^ = 0% (P = 0.90)
Asthma patients, atenolol vs. acebutolol. Mean FEV_1_	Greefhorst and van Herwaarden (1981)	0.04 [−0.59 to 0.66] P = 0.91 *I* ^2^ = 0% (P = 0.80)	0.15 [−0.67 to 0.97] P = 0.72 *I* ^2^ = 0% (P = 0.61)
Asthma patients, atenolol vs. metoprolol. Mean FEV_1_	Greefhorst and van Herwaarden (1981)	0.07 [−0.42 to 0.57] P = 0.77 *I* ^2^ = 0% (P = 0.94)	0.11 [−0.47 to 0.69] P = 0.70 *I* ^2^ = 0% (P = 0.82)
Asthma patients, metoprolol vs. acebutolol. Mean FEV_1_	Greefhorst and van Herwaarden (1981)	−0.09 [−0.82 to 0.64] P = 0.81 *I* ^2^ = 0% (P = 0.98)	Not applicable
Asthma patients, atenolol vs. metoprolol. Mean PEFR	Greefhorst and van Herwaarden (1981)	−0.02 [−0.62 to 0.57] P = 0.94 *I* ^2^ = 0% (P = 0.81)	Not applicable
Asthma patients, topical β-blockers baseline vs. after. Mean FEV_1_	Dunn et al. (1986)	−0.70 [−1.56 to −0.03] P = 0.04 *I* ^2^ = 11% (P = 0.29)	Not applicable

Abbreviations: 95% CI, 95% confidence interval; FEV_1_, forced expiratory volume in 1 second; PEFR, peak expiratory flow rate; SMD, standardized mean difference.

## 4 Discussion

### 4.1 Summary of main findings

#### 4.1.1 Effect of propranolol in asthma patients using inhaled corticosteroids

Short et al. (2013) conducted the first placebo-controlled study, yielding results opposite to those previously reported. The authors demonstrated that in patients with mild-to-moderate asthma who used inhaled corticosteroids (ICS), chronically administered propranolol had no significant effect, compared with placebo, on methacholine- or histamine-induced airway hyperresponsiveness, nor did it result in any changes in the Asthma Control Questionnaire (ACQ) or the Quality-of-Life Questionnaire (AQLQ). This study provided new insights, as previous studies on this issue had been conducted in patients not using ICS.

#### 4.1.2 Evaluation of nadolol tolerability in asthmatic patients

When considering the results from studies on non-selective β-blockers, it is essential to emphasize the findings reported by Hanania et al. (2008). The authors conducted a prospective, open-label pilot study to examine the safety and effects of nadolol in patients with mild asthma. The authors observed good tolerability of nadolol administered in gradually increasing doses to patients with mild asthma. Additionally, it was suggested that nadolol may have a beneficial effect on airway hyperresponsiveness. However, the authors themselves emphasize the limitations of this study, and these results should be interpreted with caution.

#### 4.1.3 Assessment of topical β-blockers in asthmatic patients

##### 4.1.3.1 Findings reported by Kido et al.

Kido et al. (2022) conducted a retrospective longitudinal cohort study to evaluate the association between the application of topical β-blockers and subsequent asthma attacks in patients with glaucoma and asthma. However, the authors did not draw clear conclusions.

##### 4.1.3.2 Findings reported by Hepsen et al.

In a prospective and single-masked study, Hepsen et al. (2004) showed that topical timolol administration caused a significant decrease in pulmonary function in patients with asthma.

##### 4.1.3.3 Findings reported by Morales et al.

Morales et al. (2016) drew similar conclusions to those found by Hepsen et al. (2004) in their population-based study and meta-analysis of clinical trials. Additionally, the authors presented interesting observations that despite the availability of safer agents, topical β-blockers are still frequently prescribed to patients with asthma and ocular hypertension.

##### 4.1.3.4 Findings reported by Shoene et al.

Schoene et al. (1984) conducted a randomized, double-masked, crossover study, revealing that timolol may adversely affect patients with reactive airway disease, and betaxolol caused airflow reduction in the same patients. However, only one article provided results that were included in the meta-analysis (Dunn et al., 1986), which showed that topical betaxolol and timolol statistically significantly reduced FEV1.

##### 4.1.3.5 Assessment of topical β-blockers in asthmatic patients–Overview

Although administered topically, β-blockers such as timolol eye drops can lead to measurable systemic effects, including reductions in FEV_1_. This is primarily due to systemic absorption through the conjunctival vessels and nasolacrimal drainage into the nasal mucosa, which bypasses hepatic first-pass metabolism (Kido et al., 2022). As a result, plasma concentrations can reach levels comparable to low-dose oral β-blocker therapy. Additionally, the degree of airway response may be influenced by patient-specific factors, such as underlying asthma, bronchial hyperresponsiveness, or genetic polymorphisms that affect β-adrenergic receptors. Even small systemic β2-blockade in susceptible individuals can result in clinically significant bronchoconstriction (Kido et al., 2022; Hepsen et al., 2004). These findings underscore the importance of caution when prescribing topical β-blockers, especially in patients with a history of airway disease.

#### 4.1.4 No significant differences in respiratory impact among β-blockers

The meta-analysis aimed to compare FEV_1_ and PEFR outcomes across different β-blockers (e.g., atenolol vs. acebutolol) to identify those with the most favorable respiratory safety profile. However, no statistically significant differences were found between the β-blockers analyzed, suggesting they may have a comparable impact on FEV_1_ and PEFR values.

Similar observations were described by Philip-Joet et al. (1986), who noted that FEV1 was significantly lower after both atenolol and bevantolol administration; however, there was no significant difference between the effects of the two β-blockers on FEV1.

#### 4.1.5 Propranolol and pulmonary function in asthmatic patients

Among non-selective β-blockers, propranolol has been extensively studied. Myers et al. (1996) and S Short et al. (2012) demonstrated that propranolol, at a provocative concentration, causes a reduction in FEV1 in asthmatic patients. However, Short et al. (2014) showed, in a *post hoc* analysis of a double-blind, randomized, placebo-controlled trial, that no bronchoconstriction was observed in asthma patients during the administration of propranolol in the presence of tiotropium.

#### 4.1.6 Cardio-selective vs. non-selective β-blockers: subgroup analyses and clinical implications

We also included studies that included both cardio-selective (Benson et al., 1978; Decalmer et al., 1978; Ellis et al., 1981; Greefhorst and van Herwaarden, 1981; Lammers et al., 1984; Lawrence et al., 1982) and/or non-selective β-blockers (Benson et al., 1978; Decalmer et al., 1978; Ellis et al., 1981) in our review and meta-analysis, which allowed us to perform subgroup analyses. Our meta-analysis, similar to the independent results of the included studies, showed that FEV1 results are dependent on the type of β-blocker due to its selectivity. We also included non-placebo-controlled studies comparing FEV1 at baseline and 2 h after administration of cardio-selective β-blockers (Ruffin et al., 1982; Schindl et al., 1986). Our meta-analysis did not reveal a statistically significant decrease in FEV1 following the administration of cardio-selective β-blockers. Our observations may be supported by the conclusions from the study by Morales et al. (2017). The authors of the population-based nested case–control study demonstrated that cardio-selective β-blockers are not associated with a significantly increased risk of moderate or severe asthma exacerbations in patients with asthma and cardiovascular diseases (CVDs). Additionally, they suggest that cardio-selective β-blockers may be considered for use when indicated. This opinion can be supplemented by the conclusion from their previous study (Morales et al., 2014) that the administration of β-blockers in asthma can be based on an individual evaluation of risk in patients.

#### 4.1.7 Evaluation of peak expiratory flow rate in included studies

In the meta-analysis, we also pooled PEFR results from the three included studies (Greefhorst and van Herwaarden, 1981; Lammers et al., 1984; Lawrence et al., 1982). The results of these studies, analyzed independently, show lower values for PEFR after the use of cardio-selective β-blockers; however, we did not obtain a statistically significant result in the meta-analysis.

### 4.2 Comparison with previous systematic reviews and meta-analyses

#### 4.2.1 Extending prior findings: subgroup insights into β-blocker use in asthma

The issues related to β-blockers and asthma have been addressed in several important systematic reviews and meta-analyses (Morales et al., 2014; Huang et al., 2021; Bennett M. et al., 2021). Morales et al. (2014) evaluated changes in respiratory function after acute exposure to β-blockers in patients with asthma. Additionally, the authors assessed β_2_-agonist response to acute β-blockade. Similar to our meta-analysis, they showed that although cardio-selective β-blockers are better tolerated, they are not entirely risk-free. In addition, they observed that β-blocker-induced bronchoconstriction partially responded to β_2_-agonist action. However, this response was weaker with non-selective β-blockers compared with selective ones. They also concluded that using the lowest possible dose of β-blockers with greater β_1_-selectivity reduces the risk of acute β-blocker exposure in asthma. Our meta-analysis supplements this important study with a subgroup analysis, which allows us to thoroughly confirm and complement the observations described by Morales et al. (2014). We demonstrated that the type of β-blocker (cardio-selective or non-selective) has an impact on FEV1 results in asthma patients. Moreover, we observed that in both the selective and non-selective β-blocker subgroups, the FEV1 was consistently lower than in the placebo group. However, patients using cardio-selective β-blockers achieved a smaller decrease in FEV1 than patients using non-selective β-blockers. Our analysis indicates that both selective and non-selective β-blockers are associated with a reduction in FEV_1_ in patients with asthma; however, the reduction was consistently more minor in those using cardio-selective agents. This finding supports previous evidence, suggesting that people with asthma may be better able to tolerate cardio-selective β-blockers. Despite these observations, we do not advocate a universal recommendation to always use selective over non-selective β-blockers in all clinical settings. The choice of β-blocker should remain individual, depending on the clinical indication, asthma severity, and overall cardiovascular risk. However, in general, when β-blocker therapy is necessary in patients with asthma, cardio-selective agents should be preferred, provided that patients are closely monitored for potential respiratory side effects.

#### 4.2.2 Extending prior findings: comparison with previous network meta-analysis

The study by Huang et al. (2021) represents the most up-to-date network meta-analysis (NMA) of asthma exacerbations following the administration of β-blockers. This study assessed the risk of asthma attacks with different β-blocker treatments in the general population and a population of asthma patients. The authors showed that timolol and propranolol were associated with a significantly higher risk of asthma attacks, especially in patients with a history of asthma at baseline. They concluded that timolol and propranolol should be avoided in patients at risk for asthma. These conclusions are consistent with the results of our meta-analysis, as demonstrated through the interpretation of FEV1 results, which show that non-selective β-blockers are less well tolerated in patients with asthma. In this study, we employed a classic meta-analysis based on direct comparisons of interventions, which enabled a precise assessment of effects while minimizing methodological complexity. In contrast, the work of Huang et al. (2021) used the NMA approach, integrating both direct and indirect comparison data within a multi-arm analytical framework. NMA is an interesting and evolving statistical method that enables the simultaneous comparison of multiple interventions, including those that were not directly compared in individual studies. However, it requires fulfilling several additional assumptions, such as consistency between direct and indirect results and homogeneity of the study populations across the individual arms of the network. In practice, this may lead to an increased risk of interpretation errors, especially when the number of studies is limited or there is significant heterogeneity. In turn, the classic meta-analysis we used, based solely on direct comparisons of the intervention with the control group, is characterized by greater transparency and a lower risk of errors resulting from data inconsistency. This approach, although limited to the two arms compared, allows for a more unambiguous interpretation of the results and greater precision in estimating the effects within the available data. Therefore, our meta-analysis can be seen as an extension of Huang et al.'s (Huang et al., 2021) study, providing a different perspective on the issues examined through the use of a distinct analytical method. Because the choice of method should depend on the structure of the available data and the aim of the study, this study employed a classic meta-analysis, which was the most adequate and reliable tool for assessing the issue under study while maintaining rigorous criteria for selecting studies and focusing on direct comparisons. Our primary objective was to assess changes in respiratory function following β-blocker administration in asthma patients. This approach distinguishes our study from the meta-analysis by Huang et al. (2021), who used the incidence of asthma attacks following β-blocker treatment as their primary outcome, compared with a control condition in patients with or without a history of asthma. In our selected studies, due to the established PICOT criteria, we were unable to include studies from which we could pool data for comparisons of the occurrence of asthma attacks.

#### 4.2.3 Summary of evidence on cardio-selective β-blockers

The safety of cardio-selective β-blockers in asthma was also assessed in a comprehensive review by Bennett M. et al. (2021). According to the data collected in the review process, the authors concluded that the use of cardio-selective β-blockers is not associated with an increased risk of asthma exacerbations. Additionally, an attempt was made to search the databases for incidents of death due to asthma after the use of cardio-selective β-blockers, and only one such report was found. However, the authors suggest that the reluctance to use cardio-selective β-blockers in patients with asthma is not based on these reports. The studies described in this systematic review and meta-analysis mostly yielded important findings.

Bennett M. R. et al. (2021) assessed the impact of regular bisoprolol on the response to salbutamol in asthma. The authors made an important observation that in patients with mild asthma, the bronchodilatory response to mannitol-induced bronchoconstriction during regular use of bisoprolol (a cardio-selective β-blocker) is non-inferior to placebo after administration of salbutamol.

### 4.3 Future perspectives: β-blocker safety in asthma patients—further studies on mortality and combination drug therapy needed

Despite the growing number of studies evaluating the effect of β-blockers on lung function, including FEV_1_ (Morales et al., 2014), in patients with asthma, the impact of this class of drugs on mortality in this patient group remains under-researched. Data on long-term use and its potential impact on key endpoints, such as mortality, are limited. In contrast to COPD, where a beneficial effect of β-blockers on survival has been demonstrated, as well as in patients with concomitant cardiovascular disease (MacNee, 2019; Du et al., 2014; Kubota et al., 2021), the situation in patients with asthma is less clear (Tiotiu et al., 2019). Bennett M. et al. (2021) reported data on cardio-selective β-blockers causing asthma-related deaths. In COPD, the use of β-blockers—especially cardio-selective ones—is considered safe and even recommended in guidelines (Global Initiative for Chronic Obstructive lung disease - GOLD, 2024). In contrast, in patients with asthma, concerns about the risk of bronchospasm and decreased lung function still limit the routine use of these medications (Morales et al., 2014).

Asthma is commonly managed with a combination of inhaled corticosteroids (ICS) and long-acting β_2_-agonists (LABA), which form the cornerstone of maintenance therapy in moderate to severe cases (GINA, 2024). However, to the best of our knowledge, there is a notable lack of clinical studies specifically evaluating the safety and efficacy of ICS/LABA regimens when co-administered with β-blockers, particularly in patients with comorbid cardiovascular conditions. This represents an important gap in the literature, as such combinations are increasingly likely in clinical practice given the aging population and the rising prevalence of both asthma and cardiovascular disease. Further high-quality, prospective studies are urgently needed to explore the potential interactions between these drug classes, their impact on asthma control, and overall safety in real-world settings. In Table 5, we provide a summary of the indications for using β-blockers in asthma and COPD based on our systematic review and meta-analysis of the GINA (GINA, 2024) and the GOLD (Global Initiative for Chronic Obstructive lung disease - GOLD, 2024).

**TABLE 5:** Summary of β-blocker indications in asthma and COPD. Systematic evidence and alignment with GINA (GINA, 2024) and GOLD (Global Initiative for Chronic Obstructive lung disease - GOLD, 2024).

Cardio-selective β-blocker			
β-blocker	Recommended dosage^a^	Recommendations/ contraindications—asthma	Recommendations/ contraindications—COPD
Acebutolol Atenolol Bevantolol Betaxolol (topical) Bisoprolol Ceriprolol Metoprolol Nebivolol	- If use is essential (e.g., due to cardiovascular comorbidities), initiate at the lowest possible dose, under specialist supervision, and monitor respiratory function closely - Due to the lack of specific dosing recommendations in GINA 2024 and GOLD 2024, fixed dosages were not included - Treatment decisions must be individualized based on clinical context (Cazzola et al., 2024)	**Recommendations:** cardio-selective β-blockers may be considered in acute coronary events, even in patients with asthma - Oral or ophthalmic β-blockers may be used in asthma patients if clearly indicated, under specialist supervision **Contraindications:** Asthma is not an absolute contraindication, but risks and benefits must be carefully weighed - Use with caution; initiate treatment only on a case-by-case basis and under close monitoring	**Recommendations** - There is no evidence that baseline treatment with β-blockers diminishes the respiratory benefits or heightens the cardiovascular risks associated with inhaled long-acting β-agonists in patients with chronic obstructive pulmonary disease (COPD) and elevated cardiovascular risk - β-blockers should be prescribed in patients with COPD who have cardiovascular indications (MacNee, 2019) **Contraindications** - COPD is not a contraindication - Use with caution under close monitoring

Non-selective β-blocker			
β-blocker	Recommended dosage	Recommendations/contraindications – asthma	Recommendations/contraindications – COPD
Carvedilol Nadolol Oxprenolol Pindolol Propranolol Timolol	Use is contraindicated in asthma and strongly discouraged in COPD due to a high risk of bronchospasm	**Recommendations** - Consider the use of selective β-blockers **Contraindications** - Non-selective β-blockers are contraindicated in patients with asthma	**Recommendations** - Consider the use of selective β-blockers **Contraindications** - Non-selective β-blockers should not be prescribed for patients with COPD

^a^ Due to the absence of official recommendations regarding specific β-blocker dosages in the context of asthma (GINA, 2024) and COPD (Global Initiative for Chronic Obstructive lung disease - GOLD, 2024), detailed dosing information has not been included in this summary.

In clinical practice, the decision to initiate β-blocker therapy—particularly in patients with obstructive airway diseases—requires an individualized assessment of the potential benefits and risks. The choice of agent, dosage, and titration schedule must be based on the patient’s clinical condition, comorbidities, and treatment tolerance (Cazzola et al., 2024).

Providing fixed dosage ranges might imply a level of safety that does not align with the necessary caution required when prescribing this class of medications in respiratory diseases. Therefore, a descriptive and qualitative approach was adopted, in accordance with current international guidelines.

In light of the increasing prevalence of cardiovascular comorbidities in patients with asthma (Tiotiu et al., 2019), the need for well-designed prospective studies seems urgent. Future randomized clinical trials should not only assess the effect of cardio-selective β-blockers on FEV_1_ and other spirometric parameters but also include long-term endpoints such as quality of life, risk of exacerbations, hospitalizations, and mortality. Additionally, these studies should investigate potential pharmacodynamic and pharmacokinetic interactions between β-blockers and inhaled medications, particularly in the context of complex treatment regimens for moderate to severe asthma. Taking these aspects into account in future studies may significantly alter the therapeutic approach and enable safer treatment of comorbidities in this patient group.

Although our meta-analysis focused on pulmonary function outcomes, such as FEV_1_, a comprehensive understanding of β-blocker safety in asthma also requires attention to clinical safety signals, including bronchospasm, asthma exacerbations, emergency department visits, and treatment discontinuation. Unfortunately, such endpoints were inconsistently reported or not reported at all in most of the included studies, which precluded a pooled analysis. This suggests that there is a need for more robust randomized clinical safety trials, particularly those monitoring adverse respiratory events, to draw more accurate conclusions about the use of β-blockers in patients with asthma. We recommend that future clinical trials systematically report adverse event rates and clinically relevant endpoints, such as treatment discontinuation or hospitalizations related to exacerbations.

### 4.4 Strengths and limitations

We included studies from a wide time range in a systematic review and meta-analysis. The two oldest studies are from 1978 (Benson et al., 1978; Decalmer et al., 1978), while the most recent is from 2022 (Kido et al., 2022). To the best of our knowledge, this is the most up-to-date and comprehensive systematic review and meta-analysis, providing a comprehensive treatment of β-blockers in asthma. However, this meta-analysis has limitations, likely related to potential confounding factors in some of the studies, such as the lack of broad age groups and one instance of significant heterogeneity in subgroup analysis. Attention should also be paid to the risk of bias, which may be caused by the nature of the included studies, which were, in some cases, non-randomized. However, the sample sizes of the included studies were similar, which limits the possibility that one study could dominate the results. In this systematic review, we included both RCTs and open-label and retrospective cohort studies. Additionally, we described population-based studies, nested studies, and *post hoc* analysis. In a meta-analysis, we primarily pool data from RCTs.

Although some analyses did not reach statistical significance, the observed effect sizes—interpreted according to Cohen’s classification (Andrade, 2020)—provide important clinical information. In the context of studies on the safety of β-blockers in patients with asthma, even minor effects can be of practical importance, mainly if they concern a population sensitive to changes in respiratory function. It is worth emphasizing that the effect size allows for assessing the potential importance of an intervention regardless of the sample size and P-value, which is strongly dependent on statistical power. In meta-analyses with a limited number of studies or small sample sizes, the lack of statistical significance does not rule out the existence of a real effect—it can only indicate uncertainty in the estimate. Therefore, interpreting the results solely through the prism of statistical significance can lead to erroneous conclusions. Considering the effect size, its direction, and the consistency between studies, a more nuanced assessment of the potential risk or benefit is possible. Therefore, conclusions drawn from this analysis should be considered not only in the context of the P-value but also in terms of their clinical significance.

A limitation of this meta-analysis is the inconsistent reporting of safety endpoints across the included studies. While FEV_1_ served as a measure of pulmonary safety, outcomes such as bronchospasm incidence, adverse drug reactions, or hospitalization rates were not uniformly reported or quantifiable for meta-analysis. Therefore, while our findings suggest that cardio-selective β-blockers may have a more favorable pulmonary profile than non-selective agents, a definitive assessment of safety requires better-quality data.

## 5 Conclusion

This systematic review and meta-analysis based on FEV_1_ assessment showed that patients with asthma may better tolerate cardio-selective β-blockers than non-selective β-blockers. We also showed that FEV_1_ value depends on the type of β-blocker used (when divided into cardio-selective and non-selective). We did not find statistically significant differences in PEFR between cardio-selective β-blockers and placebo. We did not demonstrate statistically significant differences between the compared β-blockers (for example, atenolol to metoprolol), which may suggest that they affect the FEV_1_ and PEFR value to a similar extent. Concerns about the use of β-blockers in asthma are well-founded. However, given the above premises, cardio-selective β-blockers may be cautiously considered in patients with asthma only when strong cardiovascular indications exist (such as heart failure with reduced ejection fraction or post-myocardial infarction) and with appropriate monitoring. Perhaps the risks will not outweigh the benefits of using β-blockers for CVDs co-occurring with asthma (for example, β-blockers reduce mortality and hospitalization rates, or long-term β-blocker therapy reduces reinfarction and improves survival). In such cases, if asthma is mild or well controlled, and treatment is initiated with a β_1_-selective agent at a low starting dose under close medical supervision, the potential cardiovascular benefits may outweigh the respiratory risks. At the same time, it is essential to emphasize that non-selective β-blockers remain contraindicated in patients with asthma. The decision to initiate therapy with a cardio-selective β-blocker should be made on an individual basis, weighing the risks and benefits carefully, ideally in consultation with both a pulmonologist and a cardiologist.

In the case of topical β-blockers in patients with asthma, we have shown a statistically significant reduction in FEV_1_. Therefore, caution should be paid, and less risky therapeutic options should be chosen.

## Data Availability

The original contributions presented in the study are included in the article/supplementary material; further inquiries can be directed to the corresponding author.
